# Modulation of immunity by tryptophan microbial metabolites

**DOI:** 10.3389/fnut.2023.1209613

**Published:** 2023-06-21

**Authors:** Siying Li

**Affiliations:** College of Bioscience and Biotechnology, Hunan Agricultural University, Hunan Provincial Engineering Research Center of Applied Microbial Resources Development for Livestock and Poultry, Changsha, China

**Keywords:** tryptophan, indole derivatives, immunity, AhR, microorganism

## Abstract

Tryptophan (Trp) is an essential amino acid that can be metabolized via endogenous and exogenous pathways, including the Kynurenine Pathway, the 5-Hydroxyindole Pathway (also the Serotonin pathway), and the Microbial pathway. Of these, the Microbial Trp metabolic pathways in the gut have recently been extensively studied for their production of bioactive molecules. The gut microbiota plays an important role in host metabolism and immunity, and microbial Trp metabolites can influence the development and progression of various diseases, including inflammatory, cardiovascular diseases, neurological diseases, metabolic diseases, and cancer, by mediating the body’s immunity. This review briefly outlines the crosstalk between gut microorganisms and Trp metabolism in the body, starting from the three metabolic pathways of Trp. The mechanisms by which microbial Trp metabolites act on organism immunity are summarized, and the potential implications for disease prevention and treatment are highlighted.

## 1. Introduction

Tryptophan (Trp), one of the functional amino acids (FAAs) for mammals, can synthesize proteins and is also a precursor for a range of biologically active molecules (1). Trp is metabolized via the Kynurenine Pathway, the 5-Hydroxyindole Pathway (Serotonin pathway), and the Microbial pathway. The metabolites serotonin, melatonin, indole, and its derivatives are essential in regulating organismal immunity (2, 3).

The gut possesses a complex ecosystem that provides a habitat for numerous microbiota. Over a long evolutionary period, the gut microbiota and its host have mutually benefited and maintained certain homeostasis (4, 5). Dysbiosis of the intestinal ecology will lead to dysfunction accompanied by increased intestinal permeability, immune system abnormalities, and organismal inflammation (6, 7). These effects can be mediated directly through intercellular interactions. Besides, they can also be mediated by metabolites produced by microorganisms, the environment or host molecules (8). As a veritable endocrine powerhouse, the gut microbiota generates various bioactive molecules. These bioactive compounds can exert effects both locally, within the gut environment, and distally, throughout the body, eliciting a range of responses that can impact multiple organ systems (8). Disruption in the reciprocal communication between the host and the microbiota may incite or intensify the development of disease pathogenesis (9, 10), which includes the mediation of disease through the immune system. The indole and indole derivatives, such as indole-3-acrylic acid (IA), indole-3-propionic acid (IPA), indole-3-lactic acid (ILA), indole-3-aldehyde (IAld), and indole-3-acetic acid (IAA), act as essential signaling molecules within the microbial community, mediating host-microbial crosstalk to affect the body’s immune system causing the development of related diseases (11–13). It is worth mentioning that indole and indole derivatives metabolized by Trp can act as activators of aryl hydrocarbon receptor (AhR) or pregnane X receptor (PXR) signaling to regulate host immune responses (14, 15).

While several previous studies have comprehensively explored the impact of Trp metabolism and gut microbes on related diseases and the crosstalk between the two (16–22), there remains a notable gap in the literature regarding the relationship between microbial Trp metabolites and organismal immunity. To address this issue, we present a concise summary of recent discoveries regarding the gut microbiota’s role in Trp metabolism. This encompasses the direct conversion of Trp into bioactive molecules by the gut microbiota, as well as their modulation of Trp metabolism in various pathways. Additionally, we provide an overview of the potential function of microbial-derived Trp metabolites as signaling molecules in the communication between the gut microbiota and organismal immunity, as well as their involvement in the pathophysiology of related diseases. Overall, this review aims to elucidate the complex interactions between microbial Trp metabolism, gut microbiota, and organismal immunity that which have important implications for human health.

## 2. Tryptophan metabolism

### 2.1. Tryptophan uptake, absorption, and metabolism

Tryptophan, also referred to as β-indolylalanine, is an aromatic amino acid characterized by the presence of a β-carbon attached to the 3-position of the indole group. With a chemical formula of C_11_H_12_N_2_O_2_ (23), Trp is the largest molecular weight of the standard amino acids and the least stored within the cell, making it more susceptible to a deficiency under conditions of nutrient deprivation and increased catabolism (12). Poultry, fish, dairy, soybean, and other foods have high Trp content (24). Humans consume Trp in the form of both ingested protein and gut microbial secretion, and the majority of Trp is absorbed in the intestine. Dietary habits, hormones, geographic location, and stress are some factors that affect Trp absorption and utilization (25). In addition, the simultaneous intake of other neutral amino acids or carbohydrates can also limit the absorption and availability of Trp (26, 27). Studies have shown that Trp uptake in the brain is enhanced during intense physical activity, activation of the neurosympathetic system, lipolysis, and increased plasma levels of non-esterified fatty acid (NEFA) (28, 29). The body’s deficiency of Trp and related metabolites is associated with various diseases, including neurological disorders, inflammatory bowel disease (IBD), metabolic diseases, cardiovascular diseases, and depression (11, 30).

Tryptophan, a neutral amino acid, is absorbed through apical membranes in the small intestine by amino acid transporters, including ATB0+ (SLC6A14 gene), a symporter utilizing 2Na/1Cl^−^, and B0AT1 (SLC6A19 gene), Na-dependent transmembrane protein (2, 31). It is then transported into the portal circulation through basolateral membranes via TAT1 (SLC16A10 gene), a uniporter, or LAT2-4F2hc (SLC7A8-SLC3A2 gene) and LAT1-4F2hc (SLC7A5-SLC3A2 gene), antiporters (32–34). The insertion of these transporters into the cell membrane, the modulation of their activity, and an increase in substrate supply require their association with other molecules, such as ACE2, CD98/CD147, or aminopeptidase N. Nonetheless, comprehension of the factors that affect the expression of these receptors and proteins remains insufficient. After being absorbed into the circulation, Trp binds mainly to albumin (75–90%) (30). Trp enters the liver with the blood circulation for protein synthesis, and the rest of Trp continues to reach tissue cells throughout the body with the blood circulation for protein synthesis (35). Trp that does not undergo protein synthesis will enter various metabolic pathways, mainly through Endogenous Trp metabolism, including the kynurenine and the serotonin pathway (36, 37). Within these pathways, the formation of endogenous metabolites such as kynurenine (KYN), kynurenic acid (KYNA), 3-hydroxykynurenine (3-HOK), and 5-hydroxytryptophan (5-HTP) takes place (38, 39). In addition, a portion of Trp that does not enter the circulation will be metabolized and degraded by intestinal microorganisms (Bacterial Trp metabolism) (40), also known as exogenous Trp metabolism. Notably, exogenous metabolites indole and indole derivatives are produced during this process (39).

### 2.2. Kynurenine pathway

The kynurenine pathway (KP) is the primary catabolic route for Trp. Once Trp is transported to the bloodstream from the intestine, approximately 90% of the unbound Trp, which is not used for protein synthesis, undergoes catabolism through this pathway in the liver (41, 42). The enzymatic activities involved in the KP and the availability of Trp are crucial factors affecting this process. Specifically, the initial and rate-limiting step for the production of downstream metabolites involves the oxidation of Trp to N-formyl-kynurenine (Nfk) by indoleamine 2,3-dioxygenase 1 and 2 (IDO1, IDO2) as well as Trp 2,3-dioxygenase (TDO). IDO and TDO are subject to stabilization by its substrate, leading to reduced inactivation rates (43–45). IDO and TDO are tissue specific. The IDO is present in various organs including the brain, gastrointestinal tract and liver, and is related to the body’s inflammatory response. IFN-γ, TNF-α, and IL-6, inflammatory cytokines effectively induce IDO activity. TDO is expressed almost exclusively in the liver, and the hypothalamic–pituitary–adrenal axis positively affects TDO expression through glucocorticoids, and to a lesser extent in the brain (45–47). Under the mediation of arylformamidase, N-formylkynurenine is catalyzed to kynurenine concisely. Kynurenine (KYN) can form kynurenic acid (KYNA) in the presence of kynurenine aminotransferase (KAT) (42, 48).

In addition to this, kynurenine can be formed to form 3-hydroxykynurenine (3-HOK) catalyzed by kynurenine 3-monooxygenase (KMO). 3-HOK can be converted to xanthurenic acid (XA) catalyzed by KAT. In addition, 3-hydroxyanthranilic acid (3-HAA) can also be formed under the catalysis of Kynureninase (49, 50). 3-HAA is metabolized in three ways. (1) As one of the most critical products of the KPquinolinic acid (QUIN) is formed from 3-HAA catalyzed by 3-HAA oxygenase (36). QUIN can be converted to NAD as the end product of the kynurenine-quinolinic acid pathway (17). (2) 3-HAA is catalyzed by 3-hydroxyanthranilate 3,4-dioxygenase to form 2-amino-3-carboxymuconate semialdehyde, and then in 2-amino-3-carboxymuconate semialdehyde carboxy-lyase catalyzed formation of Picolinic acid (51). (3) Conversion of 2-amino-3-carboxymuconate semialdehyde to acetyl CoA (complete oxidation) (52, 53). Among the three possible sub-metabolic pathways of KP, Trp is completely oxidized to carbon dioxide and water via acetyl CoA for the majority of reasons that we will not discuss here.

### 2.3. 5-Hydroxyindole pathway

**Figure 1:**
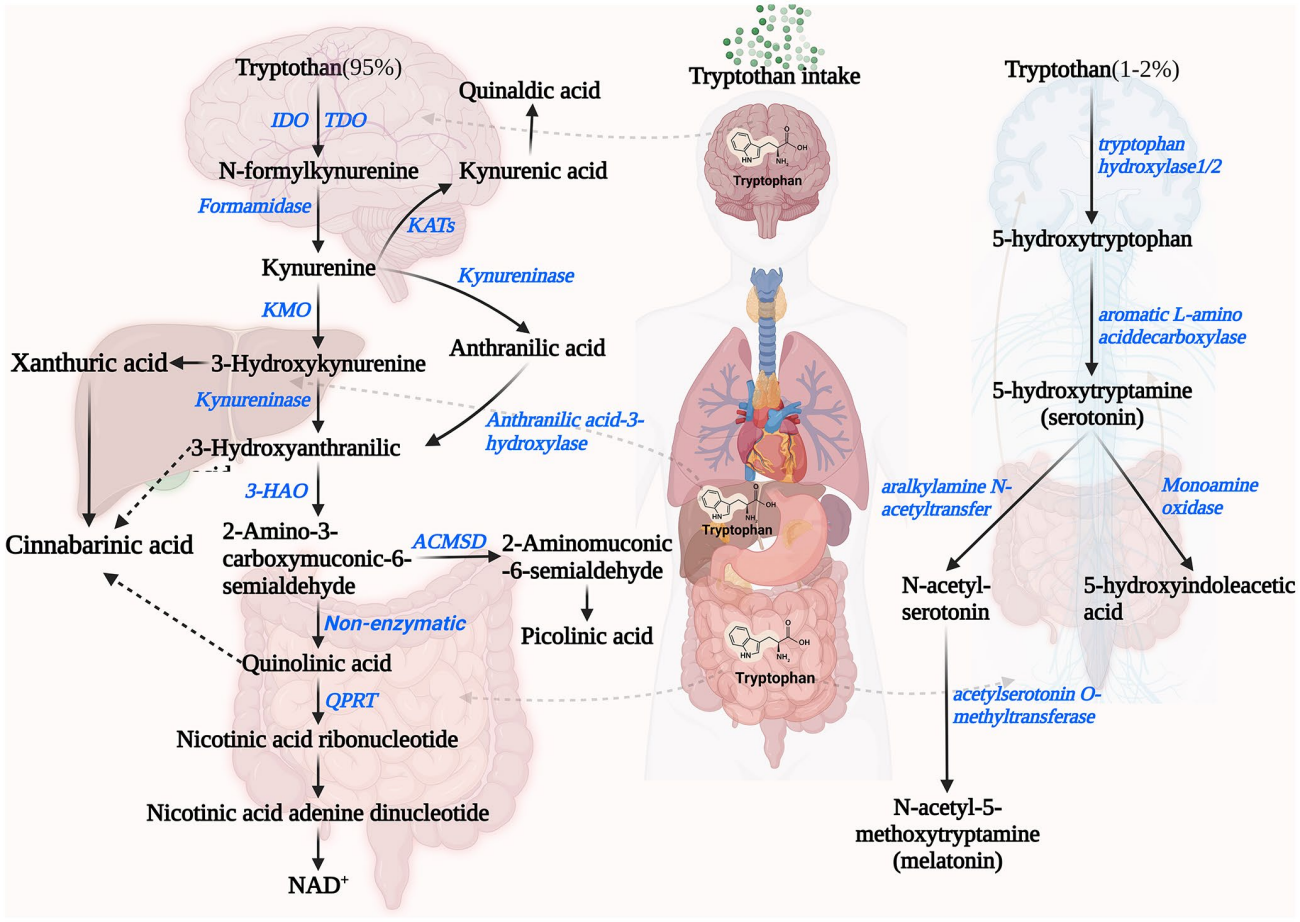
Endogenous pathways of tryptophan metabolism including Kynurenine Pathway (left) and 5-Hydroxyindole Pathway (right). IDO, indoleamine 2,3-dioxygenase; TDO, tryptophan 2,3-dioxygenase; KMO, kynurenine 3-monooxygenase; KAT, kynurenine aminotransferase; 3-HAO, 3-hydroxyanthranilicacid 3,4-dioxygenase; ACMSD, 2-amino-3-carboxymuconate-6-semialdehydedecarboxylase; QPRT, quinolinate phosphoribosyltransferase; and NAD^+^, nicotinamide adenine dinucleotides^+^.

The Serotonin Pathway is another pathway involved in the endogenous metabolism of Trp. Serotonin (5-hydroxytryptamine, 5-HT) is a monoamine molecule that is primarily synthesized in mammals through the catabolism of Trp (54). 5-HT, a key inhibitory neurotransmitter, is found in high levels in the cerebral cortex and synapses, where it is involved in mood control, regulation of sleep and pain, etc. (55, 56). In addition, 5-HT acts as a strong vasoconstrictor and smooth muscle contraction stimulator in peripheral tissues. Initially, Trp hydroxylase (TPH)-1/2 converts Trp to 5-hydroxytryptophan (5-HTP). Enterochromaffin cells located in the gastrointestinal tract express the enzyme TPH-1, and serotonergic neurons in both the central nervous system (CNS) and enteric nervous system (ENS) express the enzyme TPH-2 (17, 37, 57). Serotonin is formed from 5-HTP by the catalytic action of aromatic amino acid decarboxylase. In the intestine, TPH-1 catalyzes the production of serotonin, which can act locally or be transported by platelets to various distal sites, such as the liver, bones, and cardiovascular system (58). Although 5-HT is essential in maintaining the normal function of the central and peripheral nervous system, it cannot cross the blood–brain barrier under physiological conditions (59). 5-HT catalyzed by TPH-2 is produced in the CNS and periphery in small but significant amounts to act locally (60). Melatonin is metabolically synthesized from Trp in the pineal gland or the EC in the gastrointestinal tract (61). Trp is converted to 5-HT by the catalytic action of various enzymes. Initially, 5-HT is converted to N-acetyl-5-hydroxytryptamine catalyzed by serotonin-N-acetyltransferase, then to melatonin catalyzed by acetylserotonin O-methyltransferase (62). Besides that, Monoamine oxidase (MAO) breaks down 5-HT through catabolism, resulting in the formation of 5-hydroxyindole (5-HI) acetaldehyde. Subsequently, aldehyde dehydrogenase further metabolizes the acetaldehyde to form 5-HI acetic acid (5-HIAA), which is eliminated from the body through urine excretion (63) (Figure 1).

### 2.4. Microbiome pathways

In addition to the 5-HT pathway and KP, a small fraction of Trp enters the large intestine to be metabolized by microorganisms. The metabolic breakdown of Trp is proportional to intake, carbohydrate consumption and colonic pH (11, 64). As carbohydrate substrates are progressively depleted along the colon from proximal to distal regions, bacterial metabolism shifts from utilizing carbohydrates to proteins via fermentation. The transition leads to a notable rise in phenolic compound levels, resulting from the breakdown of Trp in the gut content, which are considerably more abundant in the distal colon compared to the proximal colon (65).

**Figure 2:**
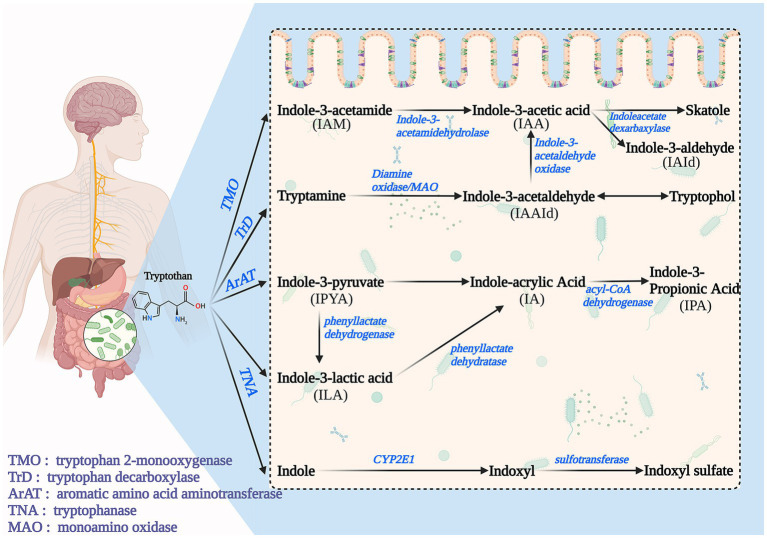
Tryptophan metabolism exogenous pathway is also known as the microbial pathway.

In Microbiome Pathways, Trp is degraded by intestinal microorganisms to indole and its derivatives, including IAA, IAld, IPA, IA, Indole-3-Acetamide (IAM), etc., which is a complex process and accompanied by many enzymatic reactions. Microbiome Pathways can be broadly classified into four pathways (Figure 2). Path I: Trp is converted to IAM (by Tryptophan 2-monooxygenase) and then to IAA (by indole-3-acetamidehydrolase) (9, 28). Path II: Trp is converted to tryptamine and then to Indole-3-acetaldehyde(IAAld) by Tryptophan decarboxyolase, Diamine oxidase, and MAO. IAAld can be converted to Tryptophol or IAA catalyzed by Indole-3-acetaldehydeoxidase. IAA can be converted to skatole (by Indoleacetate dexarbaxylase) or IAId (28). Path III: Trp gradually transforms to Indole-3-Pyruvate(IPyA; by Aromatic amino acid aminotransferase), ILA (by phenyllactate dehydrogenase), and IA (by phenyllactate dehydratase), and eventually to IPA(by acyl-CoA dehydrogenase) (9, 40). Path IV: Trp is catalyzed by Tryptophanase to indole, which can be further converted to Indoxyl sulfate (I3S) (13).

Many intestinal microorganisms are involved in metabolism, and most mediate metabolism by forming the different types of enzymes required for the pathway (66). One of the most well-studied microorganisms is *Escherichia coli* (*E. coli*). In *E. coli*, the indole pathway is regulated by a transcriptional regulator called tryptophanase (TnaA), which catalyzes the conversion of Trp to indole and pyruvate (67). Additionally, *E. coli* also possesses an indole importer (YhcY) and an efflux pump (MdtEF), which regulate the intracellular concentration of indole and control its secretion (68). Another microorganism involved in the indole pathway is *Clostridium sporogenes* (69). Trp is converted to indole by the enzyme tryptophanase in this microorganism, which is then further metabolized to IAld by an aldehyde dehydrogenase. IAld can then be oxidized to IAA by an oxidoreductase or converted to tryptamine by a decarboxylase. Several other microorganisms, including *Bacteroides thetaiotaomicron*, *Proteus vulgaris*, and *Pseudomonas aeruginosa*, have also been shown to possess enzymes involved in the indole pathway of Trp metabolism (70–73). Furthermore, *Pseudomonas aeruginosa* has been found to have a tryptophanase enzyme regulated by a two-component system, which is vital for regulating virulence (74).

## 3. Crosstalk between intestinal microbes and tryptophan metabolism

An increasing body of literature suggests that Trp catabolites, produced by the gut microbiota, play a critical role as signaling molecules in both microbial communities and communication between hosts and microbes (9). These metabolites may have a role in maintaining immune system stability within the body (75). As seen previously, bacterial Trp metabolites include indoles and indole derivatives, most of which are produced by intestinal microorganisms, including indole, IAA, IA, IPA, tryptamine, and skatole. Bacterial Trp metabolites can act as potent bioactive compounds to mediate organismal immunity. Metabolites can affect immune cells and intestinal barrier integrity by activating human receptors such as PXR or AhR, both highlighted in subsequent sections (9, 65). Different bacteria produce different enzymes to catalyze different metabolites. Since the production of some metabolites requires multiple enzymatic reactions to produce them, multiple bacterial mediators are required (Table 1).

**Table 1:** Microbiota-derived tryptophan metabolites and gut microbiota species.

Microbial tryptophan metabolites	Enzymes	References	Producers		References
Indole	Tryptophanase (TNA)	(28, 40)	*Bacteroides ovatus*	*Enterococcus faecalis*	(11, 76–80)
			*Clostridium limosum*	*Escheichia coli*	
			*Clostridium bifermentans*	*Fusobacterium nucleatum*	
			*Clostridium malenomenatum*	*Haemophilus influenza*	
			*Clostridium lentoputrescens*	*Proteus vulgaris*	
			*Clostridium tetani*	*Paracolobactrum coliforme*	
			*Clostridium tetanomorphum*	*Pseudomonas. aeruginosa*	
			*Clostridium sordellii*	*Peptostreptococcus asscharolyticus*	
			*Clostridium ghoni*	*Salmonella enterica*	
			*Corynebacterium acnes*	*Vibrio cholerae*	
			*Citrobacter koser*	…for more see (76, 81)	
Indole-3-acid-acetic (IAA)	Tryptophan decarboxylase (TrD)	(30)	*Bacteroides thetaiotaomicron*	*Clostridium sporogenes*	(69, 78, 82–85)
			*Bacteroides eggerthii*	*Clostridium perfringens*	
	Indole-3-acetaldehyde oxidase		*Bacteroides ovatus*	*Clostridium putrefaciens*	
			*Bacteroides fragilis*	*Clostridium saccharolyticum*	
	Tryptophan 2-monooxygenase (TMO)		*Bifidobacterium adolescentis*	*Clostridium sticklandii*	
			*Bifidobacterium longum subsp. longum*	*Clostridium subterminale*	
	Diamine oxidase		*Bifidobacterium pseudolongum*	*Clostridium subterminale*	
			*Clostridium bartlettii*	*Escherichia coli*	
	Monoamine oxydase (MAO)		*Clostridium difficile*	*Eubacterium hallii*	
			*Clostridium lituseburense*	*Eubacterium cylindroides*	
			*Clostridium paraputrificum*	… for more see (82)	
3-methylindole (skatole)	Indoleacetate decarboxylase (IAD)	(30, 65)	*Bacteroides thetaiotaomicron*	*Eubacterium cylindroides*	(86, 87)
			*Butyrivibrio fibrisolvens*	*Eubacterium rectale*	
			*Clostridium bartlettii*	*Megamonas hypermegale*	
			*Clostridium scatologenes*	*Parabacteroides distasonis*	
			*Clostridium drakei*	… for more see (65, 88)	
Indole-3-acrylic acid (IA)	Aromatic amino acid aminotransferase (ArAT)	(11, 65)	*Clostridium sporogenes*		(12, 69, 89, 90)
	Phenyllactate dehydrogenase (fldH)		*Peptostreptococcus russellii*		
	Phenyllactate dehydratase (fldBC)		*Peptostreptococcus anaerobius*		
	Pyruvate: ferredoxin oxidoreductase B and C (porB, C)		*Peptostreptococcus stomatis*		
			… for more see (91)		
Indole-3-propionic acid (IPA)	Acyl-CoA dehydrogenase (acdA)	(30)	*Bacteroides*	*Escherichia. coli*	(69, 89, 92, 93)
			*Clostridium botulinum*	*Peptostreptococcus asscharolyticus*	
			*Clostridium caloritolerans*	*Peptostreptococcus russellii*	
			*Clostridium paraputrificum*	*Peptostreptococcus anaerobius*	
			*Clostridium sporogenes*	*Peptostreptococcus stomatis*	
			*Clostridium cadvareris*	… for more see (94)	
Indole-3-lactic acid (ILA)	Aromatic amino acid aminotransferase (ArAT)	(30, 65)	*Anaerostipes hadrus*	*Clostridium sporogenes*	(69, 83, 95–97)
			*Anaerostipes caccae*	*Clostridium saccharolyticum*	
			*Bacteroides thetaiotaomicron*	*Escherichia coli*	
			*Bacteroides eggerthii*	*Eubacterium rectale*	
			*Bacteroides ovatus*	*Eubacterium cylindroides*	
	Phenyllactate dehydrogenase (fldH)		*Bacteroides fragilis*	*Faecalibacterium prausnitzii*	
			*Bifidobacterium adolescentis*	*Lactobacillus murinus*	
			*Bifidobacterium bifidum*	*Lactobacillus paracasei*	
			*Bifidobacterium longum subsp. infantis*	*Lactobacillus reuteri*	
			*Bifidobacterium longum subsp. longum*	*Megamonas hypermegale*	
	Pyruvate: ferredoxin oxidoreductase B and C (porB, C)		*Bifidobacterium pseudolongum*	*Parabacteroides distasonis*	
			*Clostridium bartlettii*	*Peptostreptococcus asscharolyticus*	
			*Clostridium perfringens*	… for more see (40)	
Indole-3-aldehyde (IAld)	Tryptophanase (TNA)	(98)	*Lactobacillus johnsonii*		(96, 99, 100)
			*Lactobacillus. reuteri*		
			*Lactobacillus. acidophilus*		
			*Lactobacillus. murinus*		
			…for more see (100)		
Tryptamine	Tryptophan decarboxylase (TrpD)	(98)	*Bacteroides*		(93, 101)
			*Clostridium sporogenes*		
			*Escherichia. coli*		
			*Firmicutes C. sporogenes*		
			*Ruminococcus gnavus*		
			…for more see (93)		
Indole-3-acetaldehyde (IAAld)	Diamine oxidase	(40)	*Escherichia coli*		(102)

Each indole derivative corresponds to the enzyme required for the process of synthesis of that derivative from tryptophan.

In addition to Bacterial Trp metabolism, numerous studies have shown that Endogenous Trp metabolism is also mediated by microorganisms (41, 65). Serotonin is one of the critical products of Endogenous Trp metabolism and is mainly released from intestinal chromogranin cells (103). It has been demonstrated to function as an intestinal signaling molecule, conveying signals from the gut to intrinsic or extrinsic neurons and influencing intestinal peristalsis, motility, secretion, vasodilation, and nutrient absorption (104–107). These effects suggest a potentially significant role in the regulation of gastrointestinal physiology (104). Investigations employing germ-free mice have demonstrated a noteworthy reduction in the levels of serotonin present in both the colon and blood (108). A further investigation utilizing germ-free mice colonized with specific pathogen-free fecal microbiota has shown that commensal microbiota synthesizes serotonin within the gut lumen by deconjugating glucuronide-conjugated serotonin, which is excreted via the bile duct through a β-glucuronidase-dependent mechanism (109). This discovery emphasizes the significant role of commensal microbiota in regulating gut serotonin levels, which can be modulated in multiple ways. Studies have revealed that commensal bacteria promote serotonin biosynthesis in colonic enterochromaffin cells through a mechanism involving metabolites and cell components (110, 111). *Hafnia alvei* (NCIMB, 11999), *Escherichia coli* K-12, *Lactobacillus plantarum* FI8595, *L. lactis subsp. lactis* IL1403, *Klebsiella pneumoniae* (NCIMB, 673), *Morganella morganii* (NCIMB, 10466), *Lactococcus lactis subsp. cremoris* MG1363, and *Streptococcus thermophilus* NCFB2392 have been reported to produce serotonin by expressing Trp synthetase (37, 112). Moreover, several other factors have been found to modulate the gut microbiota’s effects on serotonin production. For instance, dietary components such as fiber have been shown to stimulate serotonin production by promoting the growth of specific gut bacteria (113). Microbial biotransformation of bile acids changes secondary bile acids to deoxycholic acid inducing 5-HT synthesis (114). Similarly, alterations in the gut microbiota composition resulting from disease, medication use, or other factors can impact serotonin biosynthesis (105, 115, 116).

As the most critical enzyme in KP, IDO, the effect of the gut microbiota on IDO1 activity has been extensively studied (53, 117). Germ-free mice with a deficient intestinal microbiota reduce IDO1 activity, and antibiotic treatment of mice with established microbiota results in a similar reduction in IDO1 activity (118). It is worth noting that introducing some gut microbes (e.g., *Bifidobacterium infantis*) can normalize the kynurenine-to-tryptophan ratio (119). This is due to the ability of the intestinal microorganisms to degrade Trp into a variety of metabolites (Trp microbial pathway), thereby limiting tryptophan metabolism in the KP and serotonin pathways (59). Besides that, intestinal microorganisms indirectly regulate IDO1 activity through the metabolite butyrate, a short-chain fatty acid (SCFA), which modulates the kynurenine pathway (120). Butyrate serves as the primary energy source for enterocytes and can downregulate intestinal IDO expression through two mechanisms. First, it inhibits the expression of STAT1 (121), which reduces the transcriptional activity of IDO. Second, it acts as a histone deacetylase inhibitor to suppress IDO transcription, thereby inhibiting kynurenine production from Trp (122). These researches demonstrate that gut microbes and their metabolites are essential regulator of the KP.

## 4. AHR and PXR recognize microbial pathway tryptophan metabolites

### 4.1. Aryl hydrocarbon receptor signaling pathway

The AHR is a transcription factor that regulates various physiological and pathological processes in living organisms, including detoxification, metabolism, cell proliferation, differentiation, inflammation, and immune responses (123). AHR is typically present in the cytoplasm as part of a protein complex, which includes the AhR-interacting protein Ara9, the c-SRC protein kinase (c-SRC), the 90-kDa heat shock protein (HSP90), the 23 kDa heat shock protein (p23) and AHR, when not bound to a ligand (123). Ligand binding induces a conformational change in AHR, exposing it to protein kinase C-mediated phosphorylation and promoting its nuclear translocation, where it dissociates from the protein complex and rapidly interacts with the AhR nuclear translocator (ARNT) (124). The AhR/ARNT heterodimeric complex binds to specific DNA sequences, known as dioxin-responsive elements (DREs) or xenobiotic-responsive elements (XREs), located at 5’-TNGCGTG-3′, to regulate gene transcription (125).

**Figure 3:**
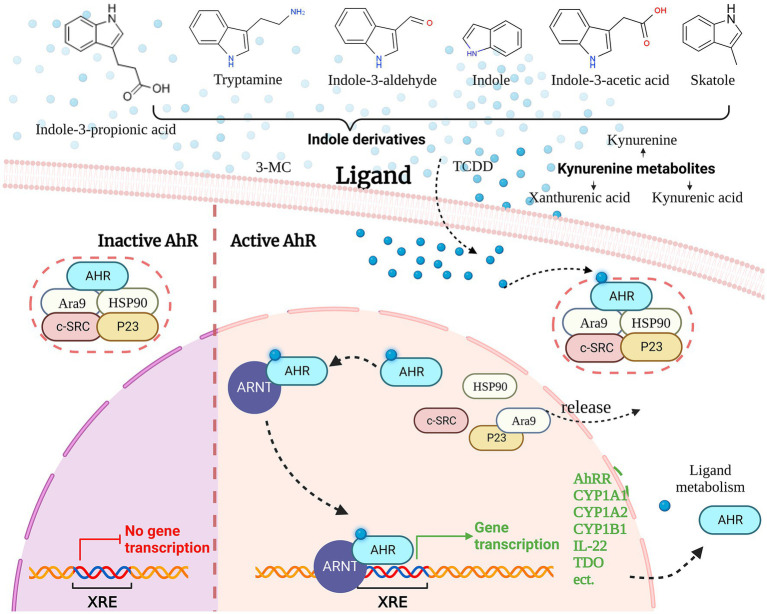
Aryl hydrocarbon receptor signaling pathway and ligands.

The most frequently mentioned genes are several genes involved in xenobiotic metabolism encoding for microsomal cytochrome P450-dependent monooxygenases. These include CYP1A1, CYP1A2, CYP1B1, and NAD(P)H-quinone oxidoreductase (126). These enzymes play a critical role in the metabolism of xenobiotics, such as environmental toxins, drugs, and carcinogens, by catalyzing their oxidation and conjugation with endogenous molecules to facilitate their elimination from the body (127). Furthermore, the AhR signaling pathway is crucial for the regulation of immunity. AhR activation regulates the differentiation, migration, and activation of immune cells, the production of inflammatory factors, and helps maintain immune homeostasis (128) (Figure 3).

### 4.2. Microbial TRP metabolites as AHR ligands

Aryl hydrocarbon receptor is a remarkably versatile nuclear receptor with the ability to bind to a wide range of diverse ligands. The ligands of AHR include endogenous ligands (Kynurenine, 6-Formylindolo [3,2-b]carbazole, and Tryptamine, etc.) and exogenous ligands (Benzo [a]pyrene and Indole-3-carbinol, 2,3,7,8-Tetrachlorodibenzo-p-dioxin, TCDD; etc.) (123, 129). AHR receptors involved in Microbiome Trp metabolism include IA, IAA, IAAld, IAId, and Tryptamine, which are important sources of receptors.

### 4.3. AHR and organismal immunity

Aryl hydrocarbon receptor is highly expressed in innate and adaptive immune cells, and recent investigations have elucidated its significant involvement in immune regulation (130). The role of AhR in regulating adaptive immunity is primarily discussed with respect to B cells and T cells (123). Experimental findings indicate that AhR-deficient mice expression display a marked increase in the overall population of B220 cells within their bone marrow compared to their wild-type counterparts (131). This effect was attributed to an increase in the number of pro/pre-B cells and immature B cells, without impacting the total number of mature B cells (131). Moreover, the potential contribution of AhR to B-cell malignancies has been examined (132). To summarize, AhR modulates B cell proliferation, differentiation, and the development of malignancies.

Aryl hydrocarbon receptor has different effects on the development and function of different T cells. AhR binding to ligands is activated to promote Tregs/Th17 cell differentiation in a ligand-specific manner (133). For example, TCDD-induced activation of AhR promotes the generation of Tregs (134); FICZ promotes the generation of Th17 cells (134). In addition to this, the AhR signaling pathway can promote the differentiation of T helper 17(Th17) cells by limiting the activation of STAT-1 and STAT-5 (135, 136), thereby reducing the expression of Th2 cytokines and modulating the Th1/Th2 balance (137).

In AhR-mediated innate immunity, Macrophages, Dendritic cells, and Lymphoid Cells (ILCs) are significantly affected. Notably, AhR exerts a negative regulatory effect on the production of proinflammatory cytokine IL-6 by inhibiting histamine production in macrophages stimulated by lipopolysaccharide (LPS) (138). Furthermore, ILCs express AhR and its genetic disruption can cause functional defects in ILCs and loss of specific subpopulations (139). Specifically, AhR is necessary for the activation of certain types of ILCs, such as liver-resident NK cells, and for the secretion of cytokines and anti-microbial peptides by ILC3s in the gastrointestinal tract (137). In addition, AhR controls DC differentiation, function and antigen presentation with profound effects on T-cell immunity (25). Overall, these studies suggest that AhR affects organismal immunity by directly and indirectly influencing the maturation and function of multiple immune cells.

In general, the AhR signaling pathway is widely recognized as a critical component of the immune defense mechanism at barrier sites (65, 140). This pathway is crucial in maintaining intestinal homeostasis by regulating various processes, including barrier stability, and immune cell function (141). However, Microbial metabolism has a dominant influence on the activity of AhR in the intestine. In the next section, the effects of gut microbial Trp metabolites on organismal immunity are explicitly described, with the AhR signaling pathway, which is activated with gut microbial Trp metabolites as ligands, occupying an important position.

### 4.4. Pregnane X receptor

Similar to the AhR, the pregnane X receptor (PXR) serves as a prominent target for the interaction with microbial tryptophan metabolites. PXR also belongs to a family of nuclear receptors (NR1I2) (142),which plays a crucial role in regulating the expression of genes involved in both drug and endobiotic metabolism, including those encoding UDP-glucuronosyl-transferases, cytochrome P450s (CYPs), drug efflux pumps, and glutathione-S-transferases (143–145). The PXR is a major regulator of the expression of the CYPs isoform, which metabolizes more than half of all human drugs (146). Therefore, PXR activation protects the body from exogenous or endogenous toxic substances and plays an essential role in the process of detoxification. PXR is expressed in a variety of cells including intestinal epithelial cells, immune cells (B cells, T cells, dendritic cells, etc.), and hepatocytes (147, 148). Activation of PXR occurs upon binding of ligands to its ligand-binding domain (LBD), leading to the formation of a heterodimeric complex with the retinoid X receptor (RXR), subsequently initiating gene transcription. PXR serves as an essential regulator of intestinal epithelial barrier function, and PXR deletion or PXR low expression decreases intestinal homeostasis and increases intestinal inflammation (149, 150).

The microbial Trp metabolites indole and indole derivatives including skatole, IAA, and IPA have been extensively studied as PXR ligands. IPA activates its receptor PXR by binding directly to the genomic region of PXR or by indirectly crosstalk with other transcription factors, such as AHR (151). These transcription factors control many genes involved in transport, inflammation, apoptosis, and oxidative stress (152). For example, PXR reduces the secretion of proinflammatory cytokines and controls inflammation by inhibiting the NF-κB signaling pathway (153, 154). In Addition, indole and IAM induced PXR regulatory genes CYPs and MDR1 in human intestinal cancer cells. I3S exerts cytostatic properties via the PXR in breast cancer models (151, 155). Clarifying the microbial Trp metabolite mediated PXR pathway would further shed light on the mechanisms that govern the metabolic cross-talk between host and microbiome with regard to Trp.

## 5. Microbial tryptophan metabolites and immunity

**Figure 4:**
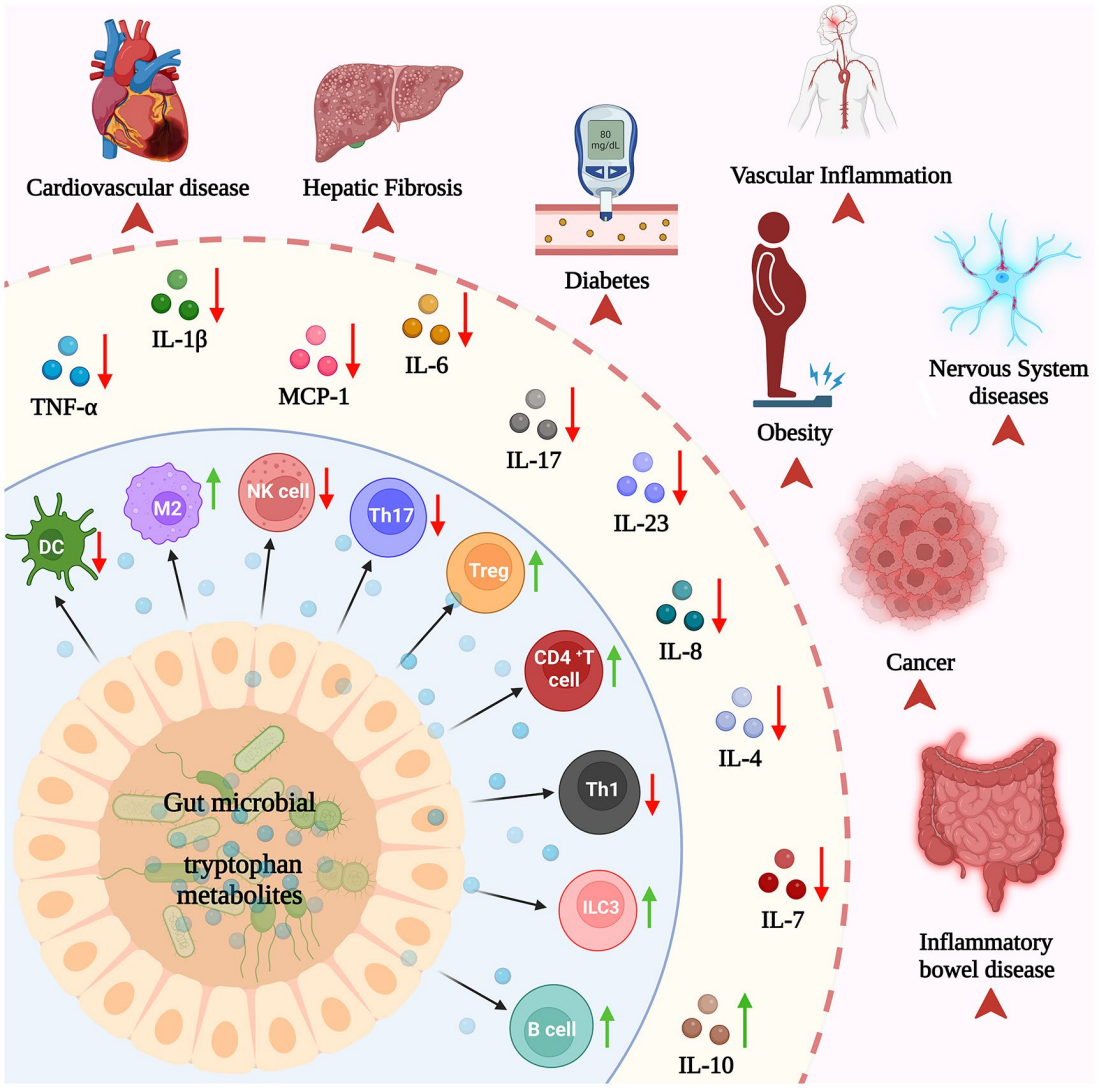
Microbial tryptophan metabolites mediate immune responses and relevant diseases.

Recent studies have highlighted the crucial role of Trp catabolites produced by the gut microbiota in serving as signaling molecules within the microbial community and host–microbe interactions (65). In turn, this significantly contributes to maintaining immune system homeostasis in the body (Figure 4).

### 5.1. Regulation of Trp metabolites from gut microbiota in the innate immunity

The innate immune system consists of skin and mucous membranes, and immune cells (neutrophils, dendritic cells, macrophages and NK cells, etc.) (156). Non-specific immunity plays a vital role in the immune system as it rapidly responds to a broad spectrum of pathogens even without prior exposure (157). In this section, the effects of microbial Trp metabolites on non-specific immune cells and the regulation of cytokine (produced by non-specific immune cells) expression are discussed.

The microbial Trp metabolite indole and its derivatives can directly regulate the growth and differentiation of non-specific immune cells (65). For example, IAld and IAA have been shown to promote the differentiation of monocytes into dendritic cells, whereas IAA also enhances the phagocytic activity of human neutrophils and macrophages (158, 159). Moreover, indole derivatives also facilitate the differentiation of tolerogenic dendritic cells, leading to the generation of regulatory T cells that suppress the inflammatory response. These notable effects are mediated through the activation of the AHR in dendritic cells, thereby triggering the upregulation of genes associated with tolerogenic dendritic cell functionality and the production of immunomodulatory cytokines (160–162). NK cells are also a critical component of the innate immune system that is involved in defending against viral infections and tumors. Several recent studies have revealed that IAld can activate human NK cells and enhance their ability to kill cancer cells (163–165). Moreover, tryptamine has been found to activate the AHR pathway in NK cells, resulting in an augmented cytotoxicity toward tumor cells (166–168). Overall, these studies suggest that microbial Trp metabolites have a major impact on the formation and function of many non-specific immune cells and might be involved in the regulation of immune responses.

In addition to direct modulatory effects on non-specific immune cells, numerous studies have shown that microbial Trp metabolites can mediate the inflammatory response by modulating the expression of cytokines during the infection phase, with a particular emphasis on the pivotal role played by macrophages (167, 169). Macrophages, derived predominantly from bone marrow monocytes, represent a critical subset of immune cells in the body (170). As one of the most important parts of innate immunity, M1 macrophages can be induced by IFN-γ, LPS or FFA to promote inflammatory responses, kill intracellularly infected pathogens and fight tumors by releasing inflammatory cytokines such as IL-1, IL-12, and TNF (171). M2 macrophages are capable of synthesizing and releasing anti-inflammatory factors (TGF-β, IL-10, etc.), immunosuppressive factors and various tumor-promoting cytokines, which can suppress inflammatory responses and promoting tumor cell growth and metastasis (172). Numerous studies have shown that microbial Trp metabolites can suppress the inflammatory response in macrophages during the infection phase. In patients with NAFLD, IAA attenuated the effect of TNF-α secreted by macrophages on bile acid metabolism, which reversed the ratio of CDCA to CA increased by FFA and macrophage inflammatory factors (123, 173). Similar results were found in the liver of mice, where IAA attenuated HFD-induced hepatotoxicity in a dose-dependent manner. It inhibited the expression of inflammatory factors such as IL-1β, IL-23, TNF-α, MCP-1, IL-17A, and IL-6, while elevating the level of the anti-inflammatory factor IL-10. The reduced pro/anti-inflammatory cytokines ratio was also reduced (14, 174, 175). In addition to this, IPA can reduce serum inflame, matory factors TNF-α and IL-1β (176). Similarly, indole has a similar function while decreasing the inflammatory cytokine expression and increasing the anti-inflammatory cytokine expression (177). Collectively, these investigations have shown that indole and indole derivatives of microbial Trp metabolites such as IPA and IAA can reduce the expression of proinflammatory factors in a dose-dependent manner and help regulate the inflammatory response.

The regulation of cytokines by microbial Trp metabolites is mainly mediated through AHR. It was found that macrophage TNF expression was reduced in response to co-stimulation by LPS and the AhR ligands IAld and IAA. In which IAld leads to a decrease in inflammatory vesicle pathway and IL-6 signaling capacity that reduces TNF transcription (178); IAA neutralizes free radicals, thus attenuating the inflammatory response of RAW264.7 macrophages to LPS and increasing IL-8 signaling (179). Similarly, indole-3-methanol suppressed the generation of LPS-mediated inflammatory cytokines such as IL-6 in bone marrow-derived macrophages (BMM) of mice (180). Tryptamine decreased the mRNA expression levels of IL-7 and IL-6 in LPS-stimulated RAW 6.264 cells via AHR (175, 181). Microbial Trp metabolite ligands of AhR affect the metabolic and immunomodulatory processes of nonspecific immune cells. Pathways such as oxidative phosphorylation, fatty acid β-oxidation or amino acid degradation are upregulated in most cases.

### 5.2. Regulation of Trp metabolites from gut microbiota in the adaptive immunity

Apart from innate immunity, microbial Trp metabolites are believed to have a significant impact on adaptive immunity. Specifically, the focus is on the role of T cells in the adaptive immune response, which recognize and respond to specific antigens (182). Upon activation, T cells differentiate into different subtypes, such as Th cells, cytotoxic T cells, and regulatory T cells, each with a distinct function in regulating the immune response (183). Several studies have reported that indole derivatives can regulate the differentiation, activation, and proliferation of T cells, thereby modulating their function.

Indole derivatives can regulate T-cell differentiation by modulating the expression of cytokines and transcription factors. For example, the indole derivative Indole-3-carbinol (I3C) has been shown to promote Th1 cell differentiation by upregulating the expression of interferon-γ and T-bet transcription factors (184). This is crucial in the defense against the intracellular pathogens (e.g., viruses and bacteria) (185, 186). Moreover, I3C inhibit the differentiation of Th2 cells by downregulating the expression of interleukin-4-related genes and GATA3 transcription factors (184), which mediate the development of allergy and asthma (186, 187). Another indole derivative, Indole-3-propionic acid (I3S), has also exhibited the ability to regulate T-cell activation and proliferation. I3S suppresses the expression of CD25 and CD69 surface markers (188), which are critical indicators of early T-cell activation and proliferation (189, 190). Besides, I3S reduced the frequency of IL-4-producing CD4 T cells and inhibited Th2 differentiation (191). This effect was attributed to the suppression of STAT5 and STAT6 phosphorylation, which are transcription factors involved in Th2 differentiation. I3S can also induce T cell apoptosis, or programmed cell death, by upregulating the expression of pro-apoptotic proteins (192). Other indole derivatives, IAld and IPA, have also been shown to modulate immune responses by regulating T-cell function (193, 194). IAld induces T-cell apoptosis and inhibits T-cell activation by regulating the expression of pro-and anti-apoptotic proteins (195).

Most notably, T-regulatory (Treg) and T-helper 17 (Th17) cells are key T-cell lineages that predominate at mucosal sites, including the gastrointestinal tract (196). Tregs play a pivotal role in inducing immune tolerance via cytokine secretion and modulation of antigen-presenting cell or effector lymphocyte function, making them critical cellular determinants of immune homeostasis in most tissues (197). In the thymus, “natural” Tregs (nTregs) develop when T lymphocytes tightly bind to self-peptide–MHC complexes on thymic stromal cells, and FOXP3 expression is induced. In the periphery, “induced” Tregs (iTregs) arise when Antigen-presenting cells activate Th cells in the presence of IL-2 and TGF-β (198). Autoimmune disorders and IBD are associated with dysregulation of the Treg population in circulation and afflicted tissue (196). Th17 cells develop following the activation of Th cells in the presence of inflammatory cytokines like IL-1β, IL-6, and IL-23, coupled with TGF-β. Mouse models have demonstrated the essential role of Th17 cells and their secreted cytokines in the pathogenesis of IBD, with elevated levels found in affected individuals (199). Notably, the Th17 response and inhibition of Tregs are considered significant contributors to the development of aberrant inflammation in the GI tract (200). Numerous studies have shown that the microbiota Trp metabolite indole and indole derivatives modulate Treg/Th17 differentiation, suggesting that the persistence of indole promotes tolerance and suppresses colonic inflammation (201). For example, I3C treatment decreases the occurrence of colitis by reducing Th17 cells and increasing Tregs (202). Besides that, I3C increased the production of CD4Foxp3 cells in mice and ultimately reduced CD4 IL-17 cells that induce neuroinflammation (203). IAA and IPA promote Treg differentiation and function by upregulating the expression of Foxp3 and other Treg-related genes (14, 40, 204). IAA also promotes Tregs differentiation by activating the AHR pathway (14).

The AhR pathway has been described in detail above as a key pathway involved in regulating the immune response by several ligands, including indole derivatives. For example, I3S inhibited Th2 differentiation by suppressing the phosphorylation of the related transcription factors STAT5 and STAT6 (191). However, the effect of I3S on Th2 was inhibited by the AhR antagonist α-naphthoflavone, which laterally suggests that the regulation of Th2 differentiation by I3S is AhR-dependent (191). Thus, boosting circulating AHR ligands, whether from endogenous or exogenous sources, is crucial for managing inflammatory diseases in various tissues. Furthermore, *L. reuteri*-produced Trp catabolites ILA or IAAld activated AhR and enhanced IL-17 production by T cells (205). This activation of AhR by ILA derived from Trp metabolism leads to the downregulation of Thpok and the reprogramming of CD4 intraepithelial lymphocytes (IELs) into double-positive (DP) IELs (96), highlighting an AHR-mediated mechanism distinct from AhR’s impact on regulatory T cells (Tr1, Tregs), intraepithelial γδ T cells and DCs (96). Moreover, tryptamine has been shown to activate mechanistic target of rapamycin (mTOR) in Treg cells *in vitro*, induce increased expression of p4EBP1 in effector memory T (Tem) cells, and enhance phosphorylated ribosomal pS6 expression in Tem cells (206), suggesting that exogenous tryptamine promotes glycolysis in TC (cytotoxic) T cells, thereby influencing the regulation of CD4 T cell function (207). Indole derivatives can modulate immune function and maintain immune homeostasis through their effects on Th1, Th2, and Treg cells as well as the AhR pathway (15, 208). Understanding the role of indole derivatives in T-cell regulation may lead to developing new therapies to treat immune-related diseases.

In addition to their effects on T lymphocytes, indole derivatives have been shown to have inhibitory and stimulatory effects on B cells. I3C can regulate B-cell function by inhibiting the production of immunoglobulins IgM and IgG in response to various stimuli. Reducing the expression of CD69, a marker of B-cell activation, induces B-cell apoptosis and inhibits B-cell proliferation (209, 210). Tryptamine has also been shown to stimulate IgA production by B cells and to activate the transcription factor AhR to regulate B cell function (211, 212). In conclusion, indole derivatives with inhibitory and stimulatory effects on B cells may have the potential as immunomodulators for treating immune diseases. However, further research is needed to fully understand the mechanisms of action of these compounds and their potential therapeutic applications.

### 5.3. Microbial Trp metabolites maintain intestinal barrier function and mucosal integrity

Intestinal microbial homeostasis and intestinal barrier integrity are closely linked with intestinal immunity. The intestinal barrier comprises the epithelial and mucus barriers, consisting of a layer of epithelial cells. Both Tight junction (TJ) and adherens junction (AJ) proteins play an essential role in this regard (213). TJ proteins, such as ZO1 and occludin, act as a protective barrier at the apical region of neighboring epithelial cell membranes (214). Their role is to prevent the unregulated diffusion of molecules between cells, thereby maintaining the integrity of the cell–cell barrier. On the other hand, AJ proteins like E-cadherin and β-catenin, located basolaterally but subjacent to TJs, play a crucial role in adhesive forces between adjacent epithelial cells (215). These proteins aid in sealing the intestinal barrier, ensuring its integrity. The intestinal barrier integrity is maintained through the collaboration of numerous factors, including mucus proteins, active molecules, and immune factors (216). However, disruptions to these factors may lead to various conditions, such as diabetes, asthma, autism, acne, allergies, and several intestinal disorders, including inflammatory bowel disease (IBD) (217, 218). A properly functioning gut-organ axis relies on a healthy intestinal microbiota structure and an integrated intestinal barrier. Disturbances to the gut microbiota composition have been associated with disruptions in intestinal homeostasis and intestinal barrier dysfunction (219). Therefore, regulating the interaction between the intestinal microbiota and the intestinal barrier presents a new approach to treating some intestinal diseases. The function of intestinal microbial Trp metabolites in maintaining the intestinal barrier and mucosal integrity has been extensively studied.

Indole derivatives, including indole-3-ethanol (IEt), IPyA, and IAld, have been shown to regulate the integrity of the intestinal barrier by modulating TJs and AJs, which together form the apical junction complex, ultimately reducing the incidence of DSS colitis in mouse models (100, 220). Treatment with IAld, IEt, and IPyA in mice inhibited the expression of TNFR1 during DSS colitis and prevented TJ and adherens junction complex (AJC) catabolism caused by activation of NF-κB by TNFα (93, 221–223). Furthermore, these metabolites protect the intestine from AJC destruction caused by proinflammatory cytokines by promoting the intestinal epithelial cells IL-10R expression (224, 225). Similarly, in the colitis model, oral administration of indole-containing capsules enhances the expression of molecules associated with TJs and AJs in colonic epithelial cells of germ-free mice, thus mitigating epithelial injury (226). Indole is known to fortify the epithelial barrier by inducing the upregulation of genes associated with the functions of TJs, AJs, actin cytoskeleton, and mucin production, among others (177). From the above studies, it can be hypothesized that microbial Trp metabolites have multiple targets *in vivo*, but their actions are synergistic. Highly relevant to the intestinal epithelium, and interacting directly with the microbiota, they work together to maintain intestinal health and immune function. Regulation of TJ by indole derivatives can be mediated by mediating gene expression. IPA and ICOOH treatment increased the gene expression of occludin, ZO-1, and MUC-2 in IECs (227, 228), and also reduced the expression of LPS-induced inflammatory factors such as IL-1β and IL-8, ultimately decreasing the permeability between the intestinal barriers. Administration of IEt, IPyA, and indole-3-carboxaldehyde increased the expression of genes such as ocludin and ZO-1, thereby enhancing the adhesion between adjacent epithelial cells (220). The findings of these studies collectively demonstrate that bacterial TRP metabolites promote TJ protein expression and reduce proinflammatory cytokine expression.

IL-22 plays a key role in maintaining intestinal epithelial integrity and antimicrobial defense, in addition to enhancing intestinal stem cells (ISC)-mediated regeneration and proliferation of epithelial cells. The primary producers of IL-22 in the intestine, a process controlled by microbiota, are innate ILCs and ILCs that express the nuclear hormone receptor RORt (229, 230). Symbiotic microbiota inhibits IL-22 production by ILCs in the healthy mouse intestine by expressing IL-25 by epithelial cells (231). In addition, the microbiota has been shown to modulate IL-22 production via indole and indole derivatives. Treatment with IAld alleviated dextran sulfate sodium (DSS)-induced colitis in mice, improved the reduction in body weight and colonic length, and restored damage to the jejunum and colon (232). IAld activation of AHR prompts lamina propria lymphocytes (LPLs), a type of Lymphoid Cells (ILCs), to secrete IL-22. IL-22 enhances intestinal epithelial regeneration and proliferation by inducing phosphorylation of STAT3 and accelerating ISC differentiation (100). Furthermore, it has been shown that IL-22-mediated STAT3 activation reduces crypt damage and stimulates crypt formation (233). Ultimately, the proliferation of intestinal epithelial cells is accelerated, restoring the damaged intestinal mucosa and barrier function.

Indole-3-propionic acid, a ligand for PXR, promoted intestinal barrier integrity by downregulating enterocyte TNF-α while it upregulated ligand-encoded mRNAs, which led to the induction of MDR1 and regulation of the epithelial nodal complex (149). Furthermore, metabolite IA reduce inflammatory response and enhance the function of the intestinal barrier by downregulating inflammation and oxidative stress genes, including CD14, CCL2, MT2A, CYBB, IL-6, and PTAFR (89).

## 6. Diseases

Bacterial TRP metabolites are critical in regulating various immune diseases, including inflammatory, cardiovascular, neurological, metabolic diseases, and cancer. This review focuses on the impact of bacterial TRP metabolites on the pathophysiology of inflammatory bowel disease (IBD), neurological disorders, and metabolic diseases.

### 6.1. Inflammatory bowel disease

Inflammatory bowel disease is a recurrent, chronic, non-specific inflammatory condition affecting the gastrointestinal tract (234). The two most commonly observed forms of IBD are Crohn’s disease and ulcerative colitis (UC) (235). IBD is known to result from the interplay of genetic and environmental factors, such as dysregulation of the mucosal immune system, disruption of the mucosal barrier of the intestine, and imbalances in the intestinal microbial community (183). Of note, recent research has demonstrated the significant involvement of dysregulated gut microbiota in the pathogenesis of IBD (12). Moreover, several studies have suggested a possible association between microbial Trp metabolism and IBD. For example, one study found that a tryptophan-free diet increased the susceptibility of mice to colitis (236). Trp indole metabolites would be selectively reduced in the serum and colon of IBD mice (237). The levels of IAA and other indole derivatives with anti-inflammatory effects were also significantly reduced in IBD (238, 239).

Recent research has indicated that there is a potential involvement of the Th17 subpopulation in the pathogenesis of colitis models induced by TNBS and DSS. Specifically, increased inflammation and Th17 subpopulations were observed in the colon and colon-associated mesenteric lymph nodes (MLN) (240–242). Notably, treatment with I3C can significantly reduce Th17 populations while increasing Tregs. Additionally, in mice with colitis treated with I3C, normal crypt formation and colonic tissue structure were maintained, while cellular infiltration was reduced (202). In parallel, the intestinal mucus layer was thinner and the amount of IPA was significantly reduced in patients with IBD, but its levels recovered with disease remission (237). In addition, IPA-treated mice showed attenuated inflammatory infiltration and reduced structural loss of epithelium and tissue. Microbial metabolites, including IPA, can help maintain intestinal homeostasis by promoting normal secretory function of goblet cells and contributing to a stable and healthy gut microbiota (243). Therefore, it can be speculated that treatment of IBD patients to normalize IPA can reduce the disease and promote intestinal homeostasis.

The most noteworthy anti-inflammatory factor in IBD is IL-10. IL-10 or IL-10 receptor deficient mice spontaneously develop severe colitis (244). During organismal inflammation, IL-10 binds to IL-10R1 and signals to reduce the proinflammatory factors production in a variety of cells, including intestinal epithelial cells (245). Functional IL-10 signaling is essential to maintain mucin production by cupped cells, which ensures mucosal barrier function and epithelial cell homeostasis (246). Mutations in IL-10 receptors have been associated with IBD and serve as a critical marker for inflammation resolution in colitis (247). Furthermore, IA has been shown to enhance IL-10 and mucin gene expression production. Thus, restoration of Trp metabolism in the gut of IBD patients leading to an increase in IA production may promote an anti-inflammatory response to produce therapeutic effects in IBD patients (89). In summary, gut microbial Trp metabolism is expected to be a therapeutic target for patients with IBD, and microbial Trp metabolites have been demonstrated laterally in the pathophysiology of other inflammatory diseases.

### 6.2. Neurological diseases

Microbial TRP metabolites can signal to the brain suggesting a potential role for metabolites in communication between the gut microbiota and the CNS (9, 248). AhR is known to be a receptor expressed not only in the gastrointestinal tract but also in CNS cells (neurons, astrocytes, and microglia). AhR reduces proinflammatory cytokine expression in astrocytes and microglia and affects the development of neurological diseases such as Parkinson’s disease (PD), multiple sclerosis (MS) (249, 250), Alzheimer’s disease (AD), and epilepsy (251, 252). Besides, AhR significantly impacts neuronal differentiation, proliferation, and survival (253). Indole derivatives, serving as ligands for AhR, including IPA and IAA, exhibit the ability to traverse the blood–brain barrier (BBB), thereby imparting particular relevance to their mediating role in the gut-brain axis (GBA). For example, indole, I3S, IPA, and IAld can activate AhR signaling in astrocytes and inhibit inflammation in the CNS (254). IPA has importance in neurological diseases due to its potent free radical scavenging ability and antioxidant capacity to protect primary neurons and neuroblastoma cells from oxidative damage (255).

Alzheimer’s disease as the most prevalent neurodegenerative disease, β-amyloid (Aβ) abnormal accumulation, and phosphorylation of tau aberrantly are widely recognized as key events leading to AD (256). Numerous studies have increasingly shown the positive effects of indoles and their derivatives in ameliorating AD pathology (257–259). Notably, in AD patients, the expression of indole derivatives IAA, ILA, and IPA was significantly reduced (260, 261). Similarly, in a study involving APP/PS1 mice with AD, indole, ILA, IAA, and I3C were found to prevent Tau hyperphosphorylation, abnormal Aβ accumulation, synaptic damage, and to promote behavioral and cognitive recovery (251). These effects are attributed to the ability of indole derivatives to elevate the levels of PSD-95 and synaptophysin, thereby mitigating synaptic damage and promoting synaptic maturation (251). Additionally, indole derivatives suppress the release of inflammatory cytokines (IL-18, IL-1β, IL-6, and TNF-α) by upregulating the expression of AhR, inhibiting the activation of the NF-κB signaling pathway, and impeding the formation of NLRP3 inflammasome. Consequently, indole derivatives contribute to the reduction of AD inflammatory responses (262). PD is the second most prevalent neurodegenerative disease after AD (263). Similar to AD, the therapeutic potential of indole derivatives for PD has been extensively investigated. For instance, I3C ameliorates lipopolysaccharide-induced neuroinflammation models of PD, delay neuronal degeneration, and improve cognitive function (264, 265). These effects are attributed to the inhibition of the NF-κB signaling pathway, decreased activity of inflammatory cytokines such as TNF-α and IL-6, and increased levels of catalase and superoxide dismutase (265). Furthermore, Pael receptors were associated with delayed proliferation of cells overexpressed in PD. IPA treatment significantly improved the proliferation of SH-SY5Y cells overexpressing Pael receptors (266). In summary, indole derivatives hold promise as potential therapeutic agents for both AD and PD. Their ability to mitigate key pathological events, including Aβ accumulation, tau phosphorylation, synaptic damage, and inflammatory responses, highlights their potential for improving the underlying neurodegenerative processes associated with these diseases (255, 260). Further research in academia is warranted to fully elucidate the mechanisms involved and explore the translational potential of indole derivatives in the treatment of AD and PD.

In contrast, recent studies have shown that *L. reuteri*, which is involved in one of the intestinal microbial Trp metabolisms, will exacerbate Experimental autoimmune encephalomyelitis (EAE) when colonized in the host intestine (267). This is due to the activation of AHR by indole derivatives such as IAA, IAAld, and tryptamine metabolites of *L. reuteri*, which promote the value-added of IL-17-producing γδ T cells and infiltration into the CNS. In parallel, production of inflammatory factors IL-17, IFN-γ, and GM-CSF increases. When reduced dietary tryptophan consumption will suppress EAE and inflammatory T cell responses in the CNS (205). In conclusion, the communication between Trp microbial metabolites and the CNS is a complex and dynamic process involving the combined effects of various receptors (e.g., AhR and PXR) and cytokines. The activation of AhR by indole derivatives to mediate inflammation in the CNS appears to act bidirectionally, but further studies are needed. These findings suggest that a better understanding of the gut-brain connection and the role of Trp microbial metabolites in this connection may lead to new therapeutic strategies for treating neurological disorders. By promoting a healthy gut microbiota and modulating Trp microbial metabolites, we may be able to positively impact brain health and prevent the development of neurological disorders.

### 6.3. Metabolic diseases

The prevalence of metabolic diseases is increasing with the increase in high-calorie diets and the decrease in exercise in humans (268). Metabolic disorders are clustered by a variety of interrelated pathological conditions, including obesity, nonalcoholic steatohepatitis (NASH), dyslipidemia, glucose intolerance, insulin resistance, hypertension, and diabetes mellitus (269). When these disorders occur together, they strongly increase cardiovascular morbidity and mortality.

Interestingly, the role of microbial TRP metabolites in metabolic diseases has been increasingly studied by scientists in recent years. A strong negative correlation exists between IAA abundance and body mass index (BMI) (270). IAA affects high-fat diet-mediated obesity through PXR and TLR4-mediated production of IL-35 B cells (271). Moreover, indole, IPA and I3S were lower in patients with type 2 diabetes and higher IPA serum concentrations were associated with a lower incidence of T2D (272). Metabolic disorders decrease the production of AHR ligands from Trp metabolites, thereby inhibiting the AhR pathway. This reduction also lowers the production of GLP-1 and IL-22, contributing to gut permeability and transfer of LPS, which results in inflammation, insulin resistance, and liver fat deposition. Recently, it has been shown that indole reduced key protein expression in the NF-κB pathway and inhibited proinflammatory factor expression (273). Besides, Indole prevents LPS-induced cholesterol metabolism alterations by increasing 4β-hydroxycholesterol hepatic levels via transcriptional regulation, which ultimately counters the adverse effects of LPS on liver. Additionally, IAA and ILA can reduce insulin resistance via aryl hydrocarbon receptors (11).

## 7. Summary

In conclusion, microbial Trp metabolites, especially those produced by gut microorganisms, significantly influence the regulation of host immunity. The metabolites produced by gut bacteria, such as indole and its derivatives, can activate the AhR and PXR signaling pathways, which play a crucial role in immune function and maintaining intestinal barrier integrity. These metabolites are involved in modulating non-specific and adaptive immunity, stimulating the generation of anti-inflammatory cytokines and enhancing the activity of immune cells. Moreover, bacterial Trp metabolites are also involved in maintaining intestinal homeostasis and mucosal integrity, thereby contributing to the prevention of inflammation and the development of immune-related diseases. Dysbiosis of the intestinal microbiota can lead to an imbalance in Trp metabolism, contributing to the pathophysiology of variety diseases (e.g., inflammatory bowel disease, neurological diseases, and metabolic diseases).

The crosstalk between gut microbes and Trp metabolism is a critical research area still in its early stages. However, the findings presented in this review suggest that targeting the gut microbiota and its metabolites may be a promising strategy for the prevention and treatment of immune-related diseases. To fully comprehend the mechanisms by which bacterial Trp metabolites impact immunity and to identify potential therapeutic targets, further research is necessary. The significance of the gut microbiota in regulating host immunity emphasizes the necessity for continued exploration of this intricate and constantly changing system.

## Author contributions

The author confirms being the sole contributor of this work and has approved it for publication.

## Funding

This study was supported by Hunan Provincial Science and Technology Department (2021JJ30008, 2020NK2004, and 2019TP2004), Double first-class construction project of Hunan Agricultural University (SYL201802003), Postgraduate Scientific Research Innovation Project of Hunan Province (CX20210654), and Science and Technology Innovation and Entrepreneurship Project for University Students of Hunan Province (2021RC1004).

## Conflict of interest

The author declares that the research was conducted in the absence of any commercial or financial relationships that could be construed as a potential conflict of interest.

## Publisher’s note

All claims expressed in this article are solely those of the authors and do not necessarily represent those of their affiliated organizations, or those of the publisher, the editors and the reviewers. Any product that may be evaluated in this article, or claim that may be made by its manufacturer, is not guaranteed or endorsed by the publisher.
